# Differences in mental health symptom severity and care engagement among transgender and gender diverse individuals: Findings from a large community health center

**DOI:** 10.1371/journal.pone.0245872

**Published:** 2021-01-25

**Authors:** Amelia M. Stanton, Abigail W. Batchelder, Norik Kirakosian, James Scholl, Dana King, Chris Grasso, Jennifer Potter, Kenneth H. Mayer, Conall O’Cleirigh

**Affiliations:** 1 Department of Psychiatry, Massachusetts General Hospital, Boston, Massachusetts, United States of America; 2 Harvard Medical School, Boston, Massachusetts, United States of America; 3 The Fenway Institute, Fenway Health, Boston, Massachusetts, United States of America; 4 Department of Psychiatry, Veterans Affairs Boston Healthcare System, Boston, Massachusetts, United States of America; 5 Department of Medicine, Beth Israel Deaconess Medical Center, Boston, Massachusetts, United States of America; National University of Singapore, SINGAPORE

## Abstract

Mental health disparities among transgender and gender diverse (TGD) populations have been documented. However, few studies have assessed differences in mental health symptom severity, substance use behavior severity, and engagement in care across TGD subgroups. Using data from the electronic health record of a community health center specializing in sexual and gender minority health, we compared the (1) severity of self-reported depression, anxiety, alcohol use, and other substance use symptoms; (2) likelihood of meeting clinical thresholds for these disorders; and (3) number of behavioral health and substance use appointments attended among cisgender, transgender, and non-binary patients. Participants were 29,988 patients aged ≥18 who attended a medical appointment between 2015 and 2018. Depression symptom severity (*F* = 200.6, p < .001), anxiety symptom severity (*F* = 102.8, p < .001), alcohol use (*F* = 58.8, p < .001), and substance use (*F* = 49.6, p < .001) differed significantly by gender. Relative to cisgender and transgender individuals, non-binary individuals are at elevated risk for depression, anxiety, and substance use disorders. Gender was also associated with differences in the number of behavioral health (χ^2^ = 51.5, p < .001) and substance use appointments (χ^2^ = 39.3, p < .001) attended. Engagement in treatment among certain gender groups is poor; cisgender women and non-binary patients assigned male at birth were the least likely to have attended a behavioral health appointment, whereas transgender men and cisgender women had attended the lowest number of substance use appointments. These data demonstrate the importance of (1) assessing gender diversity and (2) addressing the barriers that prevent TGD patients from receiving affirming care.

## Introduction

Transgender and gender diverse (TGD; see Table 1 for definitions) individuals experience significantly greater mental health symptom severity and increased substance use compared to their cisgender counterparts [1–3], but data on the prevalence of specific mental health conditions across TGD groups is limited. The few studies that have addressed TGD-specific mental health disparities have widely neglected the heterogeneity of TGD populations (e.g., [4, 5]). This approach makes it difficult to distinguish potentially critical mental health differences across subgroups, compromising efforts to determine which populations are most likely to be at increased risk for specific psychological challenges and limiting efforts to improve engagement in treatment.

**Table 1:** Key identity terms and definitions [6] used in this manuscript.

Key identity terms	Definitions
Transgender and gender diverse	An umbrella term that may be adopted by individuals whose gender identity, gender expression, or behavior is different from what is typically associated with the sex assigned at birth (e.g. transgender man, transgender woman, non-binary individual).
Non-binary	Gender identities that do not fall exclusively in man/male or woman/female categories. Some examples include genderqueer, gender fluid, agender, and bigender. Within non-Western cultures, individuals from groups such as Two Spirit people, Fa’afafine, or Hijra are sometimes considered to comprise a ‘third’ gender, but may or may not identify as non-binary or transgender.
Cisgender	Gender identity that matches social expectations of the sex they were assigned at birth (e.g., a person assigned female at birth, who identifies as a girl/woman).
AFAB/AMAB	Assigned female/male at birth. Also DMAB/DFAB (designated male/female at birth) or FAAB/MAAB (female-/ male- assigned at birth). Terms like “born female” or “natal male” are less accurate & may be considered microaggressions.

The psychological health of non-binary individuals has been particularly under-addressed, even though this group may comprise more than a third of the TGD population in the United States [7], and very few studies have assessed differences among non-binary subgroups based on assigned sex at birth [8, 9]. Non-binary is (commonly and in this paper) used as an umbrella term to describe the diverse and nuanced ways individuals experience and identify their genders outside of/not represented by “man/male” or “woman/female” (cis or trans), including, for example, bigender, agender, genderqueer, genderfluid [10, 11]. Recent research indicates that non-binary individuals have higher odds of self-reported poor health due to mental or emotional difficulties and are likely to experience more symptoms and worse mental health outcomes compared to binary transgender (i.e., transgender men and women) and cisgender individuals [8, 12–16]. These findings can be interpreted through the lens of minority stress theory [17, 18], which proposes that health disparities result from exposure to unique forms of stress, additive to the stress experienced by the general population. According to this theory, both distal (e.g., harassment, discrimination, violence) and proximal (e.g., internalized negative beliefs about the self) stressors predict poor mental and physical health outcomes in sexual and gender minority samples (e.g. [19, 20]). Over time, these stressors interact with internal processes and result in anticipation or expectation of discrimination, rejection, or non-affirmation, potentially leading to hypervigilance toward threat and pressure to conceal one’s identity to protect from harm [21].

Extending sexual minority stress theory, first proposed by Virginia Brooks [22], to examine mental health disparities among non-binary individuals, Lefevor and colleagues found that non-binary individuals face heightened and unique stressors that differentially impact their psychological well-being [12]. Relative to binary transgender and cisgender individuals, non-binary participants were harassed, sexually abused, and subjected to traumatic events at higher rates; non-binary individuals were also more likely than those with binary genders to report symptoms of anxiety, depression, and eating disorders, as well as general psychological distress. The authors suggest that structural factors may exacerbate these non-binary mental health disparities, including a lack of cultural or societal knowledge about non-binary identities and experiences, decreased receipt of preventative health services, limited access to legal resources, and systemic discrimination. Given these findings, it is important not only to include non-binary individuals in assessments of mental health disparities but also to separate them from binary transgender individuals in analyses, as they likely face distinct stressors that may not be shared by individuals in other TGD groups.

Information on linkage to and engagement in mental health treatment among gender minority individuals who meet clinical criteria for psychological and substance use disorders (SUDs) is also limited [23, 24], even though mental health services are required to access some gender-affirming procedures (e.g., surgeries). However, there is strong evidence that TGD individuals have poor access to general healthcare services [7] as well as decreased adherence to preventative screening recommendations (e.g., lower adherence to established mammography guidelines relative to cisgender individuals [25]). Other data indicates that non-binary individuals face layered barriers to primary care and gender-affirming services [14, 26]. In a small sample of genderqueer/non-binary individuals living in the San Francisco Bay area, participants often felt misunderstood by providers who approached them from a binary transgender perspective, and some chose to “borrow” a binary transgender label to receive care [27]. Other participants in this sample personally modified their prescribed healthcare because they felt that the information did not apply to them, and others went without healthcare entirely. Additional studies have reported both significantly decreased access to care (e.g., to hormone therapies [26]) and decreased wellness visit attendance [14], indicating poorer engagement with care among those with non-binary genders compared to those who endorsed binary genders. Yet, assessments of engagement in psychological and substance use treatment across TGD groups, who may be in greatest need of these services, have not yet been thoroughly conducted. Evaluating behavioral health and substance use appointment attendance among the TGD populations—particularly among non-binary individuals, who likely face increased risk for mental health concerns relative to binary TGD individuals [12]—may highlight gender-based disparities that will inform targeted efforts to engage specific groups in treatment. Indeed, high symptom severity in the presence of low or no appointment attendance presents an important opportunity to improve linkage to and retention in care.

Given the increased prevalence of mental health symptoms and the noted barriers to care faced by TGD populations, the current study contributes to the literature by: (1) comparing differences in symptom severity for common psychological (i.e., depression, anxiety) and SUDs among cisgender men, cisgender women, transgender men, transgender women, non-binary individuals assigned male at birth (AMAB), and non-binary individuals assigned female at birth (AFAB) who presented for care at an LGBTQ-focused community health center in the northeastern US; (2) across the same six groups, assessing disparities in the proportion of patients who met clinical cut-off scores indicating likely depression, anxiety, and SUDs; and, (3) among patients whose scores surpassed the clinical thresholds, assessing disparities in the percentage of individuals who attended at least one behavioral health or substance use appointment.

## Methods

The study included 29,988 patients aged 18 or older at a large community health center in Boston, MA specializing in sexual and gender minority health care [28] that completed at least one behavioral health or substance-use related electronic patient-reported outcome (ePRO) survey during a routine medical appointment between 10/1/2015-10/1/2018. Patients were informed that the surveys were voluntary and confidential. All patients who received care at the community health center were asked to complete a brief battery of self-report questionnaires prior to their appointments. The assessment battery included the Patient Health Questionnaire-9 (PHQ-9 [29]), the Generalized Anxiety Disorder 7-item questionnaire (GAD-7 [30]), the Alcohol Use Disorders Identification Test (AUDIT [31]), and the Drug Abuse Screening Test (DAST [32]). Patients completed the ePRO surveys on tablet computers using a web-based survey software designed for patient-based measures [33]. Some patients declined to complete the measures or only completed some of the measures as they waited for their providers; patients who submitted at least one PHQ-9, GAD-7, AUDIT, or DAST during the three-year period were included in the final sample.

All study data were extracted from patients’ electronic health records via Structured Query Language (SQL). The first complete PHQ-9, GAD-7, AUDIT, and DAST measures within the three-year time period were used, and any behavioral health or substance use appointments that took place during the study period were included. The Institutional Review Board at Fenway Health approved all study procedures and granted a waiver of informed consent.

The ePRO data were analyzed across six gender groups: cisgender men, cisgender women, transgender men, transgender women, non-binary individuals assigned male at birth (AMAB), and non-binary individuals assigned female at birth (AFAB). Based on the community health center’s demographic questionnaires and the ePRO data in the medical record, a two-step method (i.e., first assessing gender and then assessing assigned sex at birth) was used to assign patients to the six gender categories. For example, patients who selected male as their assigned sex at birth and gender were categorized as cisgender men. To be most inclusive of all transgender and gender diverse patients, patients were considered transgender if their responses to the questions “What is your gender?” (options were male, female, and non-binary) and “What was your sex assigned at birth?” differed. Non-binary patients were labeled AMAB if they selected male as their assigned sex at birth and non-binary as their gender or AFAB if they selected female as their assigned sex and non-binary as their gender.

### Analyses

We examined differences in mean scores on the PHQ-9, the GAD-7, the AUDIT, and the DAST with a one-way ANOVA using IBM SPSS Statistics for Windows, Version 25.0. Post-hoc analyses were conducted using a Tukey’s honestly significant difference (HSD) test. Differences in the percentage of patients who met the clinical threshold for a likely depressive disorder (PHQ-9≥10), anxiety disorder (GAD-7≥8), alcohol use disorder (AUD; AUDIT ≥8 for cisgender and transgender men, AUDIT ≥7 for cisgender women, transgender women, and non-binary individuals [31]), and SUD (DAST≥3) were examined with chi-square statistics. Differences in the percentage of patients with dual diagnoses (i.e., patients who met the threshold for a likely depressive or anxiety disorder *and* a likely AUD or SUD) were also assessed. To determine which gender groups were most likely to meet these thresholds, we conducted post-hoc z-tests at Bonferroni adjusted p-values.

Gender differences in the proportion of patients who had PHQ-9 or GAD-7 scores that met the clinical thresholds *and* attended at least one behavioral health appointment were examined with chi-square tests of independence and post-hoc z-tests. Among patients whose AUDIT or DAST scores indicated the likely presence of an AUD or SUD, gender differences in the number of substance use appointments attended were assessed with the same procedure.

## Results

### Sociodemographic characteristics

The final sample included 29,988 individuals with a mean age of 33.9 years (SD = 13.1). Sociodemographic characteristics and the number of behavioral health/appointment types by are presented by sex assigned at birth and gender in Table 2.

**Table 2:** Sociodemographic characteristics and number of behavioral health/substance use appointments attended by sex and gender category (N = 29,988).

	Cisgender Men n = 17,521 (58.4%)	Cisgender Women n = 9,288 (31.0%)	Transgender Men n = 987 (3.3%)	Transgender Women n = 1,002 (3.3%)	Non-binary (Assigned Male) n = 428 (1.4%)	Non-binary (Assigned Female) n = 762 (2.5%)	Statistic
**Age**	**mean (±SD)**						χ^2^ **(df)**^a^
	36.88 (±13.3)	30.55 (±12.0)	25.89 (±7.9)	29.19 (±10.7)	28.89 (±11.7)	25.35 (±6.9)	χ^2^(5) = 3083.1*
**Sexual orientation**	**% (n)**						χ^2^ **(df, n)**
Heterosexual	31.7% (n = 4,505)	68.0% (n = 5,778)	31.3% (n = 269)	16.5% (n = 147)	7.7% (n = 32)	1.3% (n = 10)	χ^2^ (20, n = 25,614) = 11,279.6*
Gay/Lesbian	60.5% (n = 8,596)	16.5% (n = 1,398)	17.8% (n = 153)	28.5% (n = 253)	20.2% (n = 84)	26.9% (n = 202)	
Bisexual	4.6% (n = 659)	10.8% (n = 916)	19.1% (n = 164)	28.2% (n = 251)	24.6% (n = 102)	22.4% (n = 168)	
Other	3.1% (n = 444)	4.7% (n = 403)	31.9% (n = 274)	26.8% (n = 238)	47.5% (n = 197)	49.4% (n = 371)	
**Race**	**% (n)**						χ^2^ **(df, n)**
Native American, Pacific Islander or Other	2.9% (n = 482)	3.3% (n = 292)	7.4% (n = 71)	3.7% (n = 35)	2.9% (n = 12)	1.7% (n = 13)	χ^2^ (35, n = 28,392) = 400.1*
Black	6.7% (n = 1,107)	7.6% (n = 672)	6.0% (n = 58)	5.3% (n = 50)	5.4% (n = 22)	4.8% (n = 36)	
Asian	7.6% (n = 1,245)	12.4% (n = 1,101)	4.9% (n = 47)	5.5% (n = 52)	6.3% (n = 26)	3.5% (n = 26)	
Multiracial	4.5% (n = 747)	4.9% (n = 433)	7.0% (n = 67)	7.0% (n = 66)	8.8% (n = 36)	9.9% (n = 74)	
White	70.4% (n = 12,340)	70.1% (n = 6,241)	77.7% (n = 747)	72.0% (n = 721)	74.7% (n = 307)	79.0% (n = 590)	
**Ethnicity**	**% (n)**						χ^2^ **(df, n)**
Latinx/Hispanic	19.5% (n = 1,044)	18.3% (n = 387)	18.0% (n = 39)	24.4% (n = 63)	16.9% (n = 13)	7.1% (n = 11)	χ^2^ (5, n = 8,186) = 21.1*
**Insurance**	**% (n)**						χ^2^**(df, n)**
Private	80.4% (n = 14,087)	82.7% (n = 7,679)	75.1% (n = 741)	63.6% (n = 637)	72.2% (n = 309)	78.9% (n = 601)	χ^2^(20, n = 29,987) = 391.8*
Self-pay	3.1% (n = 546)	3.3% (n = 305)	5,4% (n = 53)	8.1% (n = 81)	5.4% (n = 23)	3.9% (n = 30)	
Medicaid	9.1% (n = 1,596)	8.0% (n = 739)	13.9% (n = 137)	19.3% (n = 193)	14.7% (n = 63)	14.4% (n = 110)	
Medicare	5.9% (n = 1,042)	4.2% (n = 391)	4.6% (n = 45)	8.1% (n = 81)	6.3% (n = 27)	2.2% (n = 17)	
Other public or grants	1.4% (n = 250)	1.9% (n = 173)	1.1% (n = 11)	1.0% (n = 10)	1.4% (n = 6)	0.5% (n = 4)	
**Number of appointments attended**							
Behavioral health	3.2 (±13.3)	3.1 (±13.1)	5.2 (±16.0)	6.7 (±18.0)	6.1 (±17.0)	7.7 (±19.1)	χ^2^(5) = 772.6*
Substance use	0.2 (±3.1)	0.1 (±2.3)	0.006 (±0.1)	0.1 (±1.8)	0.3 (±3.2)	0.5 (±6.1)	χ^2^(5) = 56.7*

*denotes p ≤ 001

^a^The age and the appointment attendance data violated the assumption of equal variance, so Kruskal-Wallis tests were used to examine differences across the six groups.

### Mental health and substance use behavior severity

**Fig 1:**
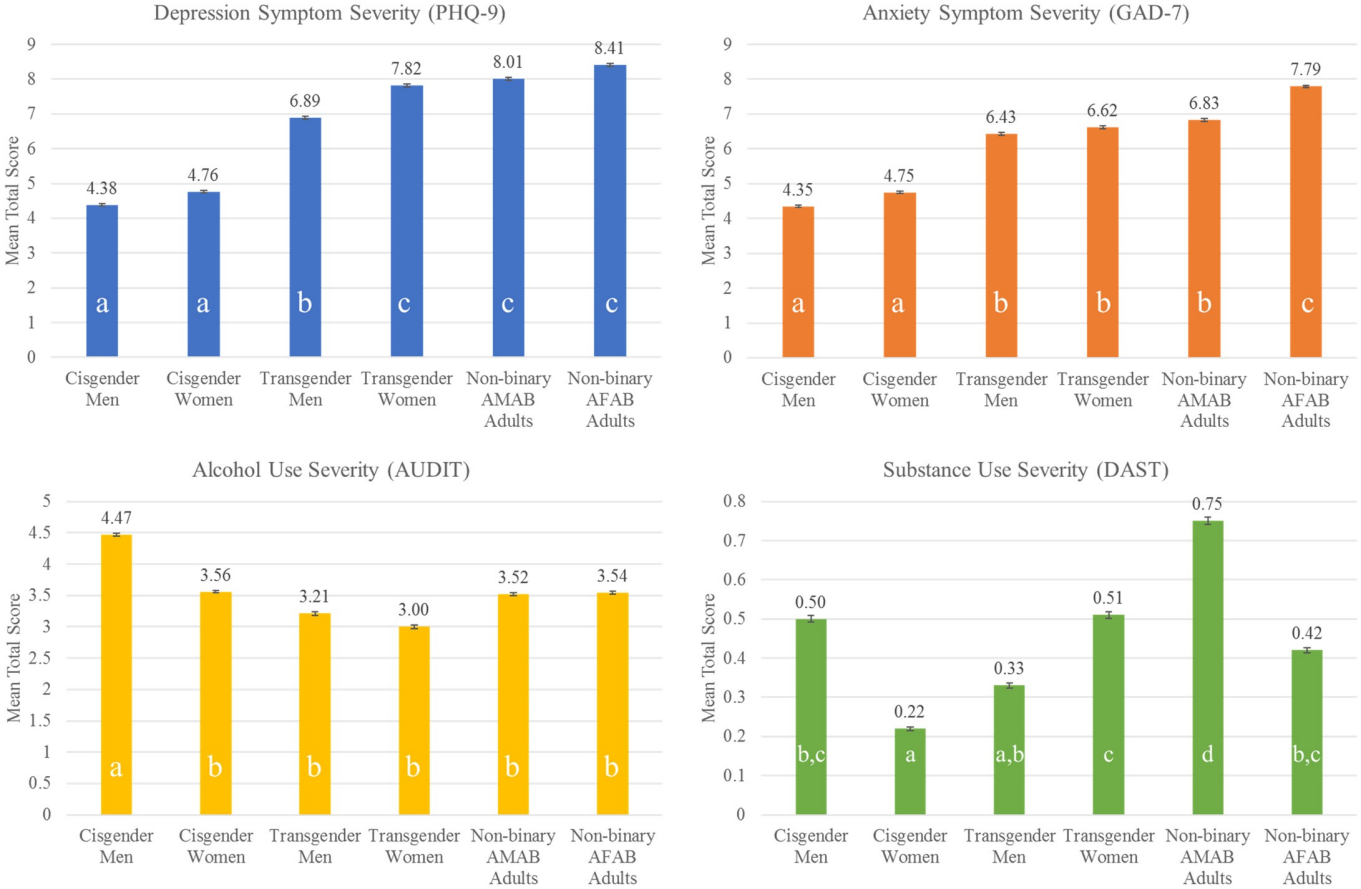
Mental health and substance use behavior severity among the gender categories. AMAB = Assigned Male at Birth; AFAB = Assigned Female at Birth. Letters embedded in each bar denote homogeneous groups (i.e. means of subgroups that have the same superscript do not significantly differ from each other) based on Tukey’s Honestly Significant Difference test significance at the p < .05 level.

Depression symptom severity differed significantly by gender category (*F*(5, 27400) = 200.6, *p* < .001), and a Tukey HSD test revealed that non-binary AFAB patients had higher severity than transgender men, cisgender women, and cisgender men (*ps* < .001; see Fig 1). Anxiety symptom severity also differed significantly by gender (*F*(5, 21463) = 102.8, *p* < .001) and was highest in non-binary AFAB patients.

Both alcohol use severity (*F*(5, 20396) = 58.8, *p* < .001) and substance use severity (*F*(5, 20092) = 49.6, *p* < .001) differed significantly by gender category (see Fig 1). Cisgender men (M = 4.5, SD = 4.5) had greater alcohol use than all other groups (*ps* < .001). Other substance use severity was significantly greater in non-binary AMAB patients than in all other groups (*p*s < .05).

Given the documented differences in age, sexual orientation, race, ethnicity, and insurance across the six gender groups (see Table 2), we ran separate linear regression models to assess the influence of gender category on symptom severity (i.e., PHQ-9 total score, GAD-7 total score, AUDIT total score, DAST severity score) when controlling for the five demographic variables; in the presence of the other variables, gender category was still significantly associated with each of the total scores.

### Likely diagnoses and linkage to care

The proportion of patients who met the PHQ-9 threshold score indicating a likely depressive disorder differed across gender category, χ^2^(5, N = 27406) = 670.3, p < .001 (see Table 3). Post-hoc z-tests revealed that more transgender women (34.6%) and non-binary patients (36.8% AFAB, 33.7% AMAB) met the clinical cut-off score, compared to transgender men (27.5%), cisgender women (16.1%), and cisgender men (14.1%). The likelihood of meeting the anxiety threshold score also differed by gender category, χ^2^(5, N = 21,469) = 397.4, p < .001, such that the proportion of non-binary AFAB patients with a likely anxiety disorder (46.1%) was significantly greater than the corresponding proportions across the five other gender groups.

**Table 3:** Likelihood of meeting behavioral health and substance use clinical thresholds and appointment attendance by gender category.

	Met clinical threshold for depression (PHQ-9≥10)	Met clinical threshold for anxiety (GAD-7≥8)	Met clinical threshold for depression and/or anxiety	Attended at least one behavioral health appointment^1^	Met clinical threshold for Alcohol Use Disorder (AUDIT≥8 or AUDIT≥7)^2^	Met clinical threshold for Substance Use Disorder (DAST≥3)	Met clinical threshold for AUD and/or SUD	Attended at least one substance use appointment^3^	Dual Diagnosis: Met clinical threshold for depression and/or anxiety *and* AUD and/or SUD
**Cisgender Men** n = 17,521	14.1%^a^ (n = 2,174)	20.3%^a^ (n = 2,447)	29.6%^a^ (n = 3,452)	41.6%^a^ (n = 1,436)	11.0%^a^ (n = 1,922)	6.2%^a^ (n = 701)	13.6%^a^ (n = 2,376)	10.1%^a^ (n = 239)	5.2%^a^ (n = 905)
**Cisgender Women** n = 9,288	16.1%^b^ (n = 1,446)	22.9%^b^ (n = 1,561)	30.7%^a^ (n = 2,164)	37.6%^b^ (n = 813)	8.8%^b,c^ (n = 818)	1.9%^b^ (n = 116)	9.5%^b^ (n = 884)	4.1%^b^ (n = 36)	4.4%^b^ (n = 410)
**Transgender Men** n = 987	27.5%^c^ (n = 262)	33.6%^c^ (n = 273)	43.6%^b^ (n = 369)	47.7%^c,d^ (n = 176)	7.2%^c^ (n = 71)	2.8%^b,c^ (n = 22)	8.9%^b^ (n = 88)	1.1%^b^ (n = 1)	4.9%^a,b^ (n = 48)
**Transgender Women** n = 1,002	34.6%^d^ (n = 329)	35.0%^c^ (n = 289)	49.6%^c^ (n = 430)	51.6%^d^ (n = 222)	8.7%^b,c,d^ (n = 87)	5.4%^a,d^ (n = 43)	10.8%^b,c^ (n = 108)	4.6%^a,b^ (n = 5)	6.4%^a^ (n = 64)
**Non-binary (Assigned Male)** n = 428	33.7%^d^ (n = 139)	38.0%^c^ (n = 135)	50.8%^c,d^ (n = 189)	41.3%^a,b,c^ (n = 78)	11.4%^a,b,d^ (n = 49)	10.4%^e^ (n = 35)	17.8%^d^ (n = 76)	6.6%^a,b^ (n = 5)	10.5%^c^ (n = 45)
**Non-binary (Assigned Female)** n = 762	36.8%^d^ (n = 274)	46.1%^d^ (n = 289)	56.4%^d^ (n = 372)	51.1%^d^ (n = 190)	11.0%^a,d^ (n = 84)	4.1%^c,d^ (n = 25)	12.9%^a,c^ (n = 98)	9.2%^a^ (n = 9)	9.1%^c^ (n = 69)

Based on results of a post-hoc z-test, each superscript letter denotes a subgroup whose column proportions do not differ significantly from each other at the p < .05 level.

^1^This category presents the behavioral health appointment attendance data for the patients who met the clinical thresholds for depression or anxiety.

^2^The higher threshold score, which is typically reserved for men, was used for cisgender and transgender men. The lower threshold score, which is typically reserved for women, was used for cisgender women, transgender women, and non-binary patients.

^3^This category presents the substance use appointment attendance data for the patients who met the clinical thresholds for AUD or SUD.

The proportions of patients who met the clinical thresholds for either a depressive or an anxiety disorder *and* attended at least one behavioral health appointment differed significantly by gender category, χ^2^(5, N = 6,976) = 51.5, p < .001 see Table 3). Higher percentages of transgender men (47.7%), transgender women (51.6%), and non-binary AFAB individuals (51.1%) attended an appointment, followed by cisgender men (41.6%), non-binary AMAB individuals (41.3%), and cisgender women (37.6%).

The likelihood of meeting the threshold AUDIT and DAST scores differed by gender category, χ^2^(5, N = 20,402) = 44.6, p < .001 and χ^2^(5, N = 20,098) = 203.0, p < .001, respectively (see Table 3). The percentages of cisgender men (11.0%), non-binary AFAB patients (11.0%), and non-binary AMAB patients (11.4%) with a likely AUD were significantly higher than the corresponding values for cisgender women (8.8%), transgender men (7.2%), and transgender women (8.7%). The likelihood of having a non-alcohol related SUD was significantly higher among non-binary AMAB patients (10.4%) than in all other gender groups. Finally, the proportion of patients who met the clinical thresholds for either an alcohol or substance use disorder *and* attended at least one substance use treatment appointment differed significantly by gender category, χ^2^(5, N = 4,069) = 39.3, p < .001 (see Table 3). Cisgender men had the highest proportion of attending at least one substance use appointment (10.1%), followed by non-binary AFAB (9.2%) and AMAB (6.6%) patients.

With respect to dual diagnoses, the likelihood of meeting both the mental health (PHQ-9 or GAD-7) and substance use (AUDIT or DAST) threshold scores differed significantly by gender category, χ^2^(5, N = 18,258) = 92.0, p < .001 (see Table 3). Post-hoc z-tests indicated that the percentages of non-binary AMAB patients (10.5%) and non-binary AFAB patients (9.1%) who met criteria for a likely mental health disorder and a likely substance use disorder were significantly higher than the corresponding percentages for transgender men (4.9%), transgender women (6.4%), cisgender women (4.4%), and cisgender men (5.2%).

## Discussion

In data extracted from the electronic medical records of almost 30,000 patients at a community health center in the Northeast US, there were significant differences in the severity of depression, anxiety, alcohol use, and other substance use symptoms by gender category, as well as significant differences in the proportions of patients across the gender groups whose PHQ-9, GAD-7, AUDIT, and DAST scores were indicative of the associated disorders. The likelihood of meeting the threshold scores for both a mental health disorder and a substance use disorder differed by gender, as did the percentages of patients who met the threshold scores and attended behavioral health and substance use appointments, though the potential association between behavioral health appointments, clinical care priorities, and gender affirming services need to be considered when interpreting the attendance findings. Though TGD populations are often studied as a homogeneous group, these results suggest that researchers and clinicians should attempt to distinguish risk for certain diagnoses and risk for poor engagement in care by subgroup and cater to specific needs.

In accordance with Lefevor and colleagues’ recent extension of minority stress theory [12], symptoms of depression were most severe among non-binary participants and transgender women, and non-binary AFAB participants had the highest anxiety symptom severity. Non-binary participants were also more likely than participants with other genders to meet criteria for dual mental health-substance use diagnoses, which can be viewed as another index of symptom severity. It has been consistently demonstrated that TGD individuals have worse depression and anxiety outcomes than cisgender men and women (e.g. [4, 34, 35]). In this sample, non-binary individuals not only had higher depression severity than cisgender individuals, they also had more severe symptoms than transgender men. Relatedly, higher percentages of non-binary individuals (AFAB and AMAB) and transgender women met the clinical threshold score indicative of a likely depressive disorder. Non-binary AFAB patients had the highest anxiety symptom severity and were most likely to meet criteria for an anxiety disorder, followed by the three other TGD subgroups, with all TGD individuals significantly surpassing cisgender individuals.

Though transgender and non-binary individuals share a minority gender identity and therefore likely experience many similar stressors, non-binary individuals may face unique additional interpersonal and structural challenges that may be associated with increased mental health symptom severity. Heightened harassment relative to binary individuals and inadequate access to health and legal resources may exacerbate existing stressors and have detrimental psychological consequences. [12, 36–38]. Recent qualitative interviews with non-binary adolescents revealed experiences of invalidation, defined as the refusal to accept one’s identity as real or true (i.e., identities are dismissed as fake, fabricated, or simply passing “phases”) [39]. In this adolescent sample, invalidation across a range of social contexts led to confusion, self-doubt, rumination, and shame, which together contributed to poor mental health outcomes. Some participants expressed concerns about not being “trans enough”, much like bisexual individuals who have reported exclusion from gay and lesbian spaces due to not being “queer enough” [40, 41]. Indeed, bisexual individuals are more likely than gay and lesbian individuals to meet diagnostic criteria for depression or anxiety [42] and to experience suicidal ideation [43]. A separate study using this data set analyzed disparities in mental health disorder severity, diagnoses, and treatment by sexual orientation; bisexual individuals and individuals who endorsed “other” as their sexual orientation (i.e., not gay, bisexual, or heterosexual), were more likely to screen positive for depression and anxiety than gay/lesbian women, gay men, and heterosexual individuals [44]. Invalidation among individuals who identify and experience their gender and/or sexual orientation in non-binary ways may be distinct from non-affirmation [39], a noted minority stressor that occurs when one’s gender is not supported or recognized by others [36]. More frequent gender non-affirmation has been associated with higher depressive and anxiety symptoms [36], and, relatedly, those experiencing affirmations of their gender identity have reduced depressive symptoms [45].

Non-binary individuals may also be less buffered by factors protective against psychological distress compared to transgender men and women. Lack of social support, for example, has been associated with detrimental impacts to TGD individuals’ mental health [35, 46]. Among genderqueer/non-binary individuals, Budge et al. found that higher social support was associated with lower depression prevalence and lower anxiety severity [46]. Given the invalidating and non-affirming experiences that many non-binary individuals face, they may also have fewer social support resources.

Our findings should also be discussed in the context of research examining differences among TGD individuals by sex assigned at birth. The few studies that have distinguished between AMAB and AFAB TGD individuals, and especially between AMAB and AFAB non-binary individuals, offer conflicting evidence. In a United States cohort of 214 TGD individuals, Newcomb et al. found that AMAB TGD individuals, inclusive of transgender women and non-binary AMAB individuals, reported worse psychosocial experiences (including higher risk for suicide, increased exposure to violence, and more substance use) relative to transgender men and non-binary AFAB individuals [8]. Conversely, among 677 TGD individuals in the UK, Rimes et al. found that TGD AFAB individuals, inclusive of transgender men and non-binary AFAB individuals, were significantly more likely than transgender women and non-binary AMAB individuals to report current mental health challenges, histories of self-harm, and childhood sexual abuse [47]. Although cross-sectional in design, these studies may point to specific mechanisms underlying the pattern of mental health and substance use related disparities among TGD individuals. Increased work in this area will be critical in rigorously assessing and integrating these findings.

Among patients whose PHQ-9 and GAD-7 scores were above the clinical thresholds, transgender men, transgender women, and non-binary AFAB individuals were most likely to have attended at least one behavioral health appointment during the study period. These data are promising; they indicate that, in high risk groups, almost half of those who need services are being connected to care; however, it is important to contextualize these findings, which originate from a community health center in the northeastern US that caters to sexual and gender minorities. Engagement with behavioral health or substance use treatment among TGD individuals may be worse in community health centers that do not specifically serve this population. Even in this setting and in this region, which includes states that have the lowest odds of transgender care refusal [48], almost half of non-binary individuals and transgender men are *not* linked to and/or did not utilize in-house behavioral health services, with even lower percentages of non-binary AMAB patients attending such appointments.

Disparities in behavioral health appointment attendance among non-binary AMAB patients—and among non-binary AFAB patients, though they were more likely to attend at least one appointment—could be explained by distal stressors and a history of negative health care or counseling experiences. Medical providers have invalidated, avoided, or overemphasized gender with non-binary patients [49], and therapists have engaged in gender generalizing (i.e., assuming a singular trans experience) and gender pathologizing (i.e., stigmatizing trans identity as the cause of all problems; [50]). For non-binary individuals, these and other negative experiences with providers may affect their engagement with behavioral health services. With this in mind, the importance of training clinicians to provide culturally competent care to TGD and sexual minority patients cannot be understated [51].

Relative to the existing literature, the alcohol and substance use results were somewhat surprising, in that alcohol use severity did not significantly differ across the four TGD subgroups. In contrast, recent studies with smaller samples have documented higher hazardous alcohol use among non-binary adults relative to binary transgender adults [14] and in non-binary and gender diverse youth compared to cisgender sexual minority youth [8]. Consistent with historical trends of high alcohol use in men relative to women [52, 53], cisgender men in this sample had the highest alcohol use across the six gender groups, and a relatively high percentage of cisgender men (11.0%) met the clinical criteria for an AUD based on their AUDIT scores. Notably, the majority of the cisgender men included here were gay, and gay men have higher rates of alcohol use compared to heterosexual men [54]. Greater proportions of non-binary patients, both AFAB (11.0%) and AMAB (11.4%), met the threshold for an AUD, indicating that non-binary individuals may be more likely to have problematic use than binary transgender individuals (corresponding rates among transgender men and women were 7.2% and 8.7%, respectively). Regarding substances other than alcohol, non-binary AMAB individuals had significantly greater use than cisgender men and all other TGD subgroups; non-binary AMAB individuals were also most likely to meet the clinical threshold for a SUD, indicating that clinicians should be particularly attentive to problematic or hazardous substance use in this subgroup.

Engagement with substance use services is poor in the general population, with only about 20% of individuals who meet criteria for an AUD ever receiving treatment [55]. At 10%, the rate of lifetime SUD treatment in a sample of binary transgender adults was even lower [56]. However, these two figures are both higher than all of the corresponding rates across all the gender groups in the current study, with the exception of cisgender men. The data were most disheartening for cisgender women and transgender individuals, with only 1.1% (n = 1) of transgender men who met criteria for a SUD (n = 88) attending at least one appointment. Though slightly higher, the percentages of transgender women (4.6%) and non-binary AMAB patients (6.6%) who attended a substance use treatment visit did not statistically differ from that of transgender men, indicating that strategies that support linkage to and engagement in care are needed for these populations. Motivators of and barriers to SUD treatment attendance among non-binary subgroups will also need to be systematically assessed and addressed, as they may differ from those of binary transgender populations.

Our findings need to be qualified in light of several limitations to the current sample and setting. First, the majority of cisgender men in the sample were gay (60.5%), whereas the majority of cisgender women were heterosexual (68.0%). Given the community health center’s focus on providing treatment for sexual and gender minority individuals, cisgender heterosexual women and men may have been less prioritized for behavioral health and substance use appointments in this setting. In addition, the sample sizes for the substance use treatment data across the TGD subgroups were small, so the results of the corresponding chi-square analyses should be interpreted with caution. However, we present these data in part *because* the sample sizes are small, with the goal of emphasizing that these individuals are in high need of services. It is also important to note the potential impact of gender-affirming care and support on the behavioral health appointment findings among TGD individuals. Behavioral health evaluations are not required to receive the gender-affirming services that the community health center provides (e.g., hormone therapy); yet, patients may have sought behavioral health services and/or evaluations to be eligible for other gender affirming services (e.g., surgical procedures) that are offered elsewhere. Unfortunately, behavioral health sessions that are used to fulfill requirements for gender-affirming surgical procedures are not coded differently in the medical record than sessions used to address other presenting concerns. Relatedly, patients may have engaged with behavioral health for support as they received these gender-affirming services; that is, depression and/or anxiety (or other mental health diagnoses that were not assessed in the current study) may have not been the focus of the appointments. This could help explain the relatively high number of behavioral health appointments attended among transgender and non-binary individuals. However, the percentage of non-binary AMAB individuals who met criteria for likely depression and/or anxiety and attended a behavioral health appointment was still relatively low, as was the percentage of transgender women who met criteria for a likely substance use disorder and attended a substance use appointment, indicating that these groups may require more attention from providers. Finally, the majority of the patients who receive care at the community health center are relatively young and identify as White. Conclusions drawn from these data may not generalize to settings that serve older, more racially diverse patients. Much of the research with TGD individuals, particularly among non-binary individuals, has been conducted with younger samples, as they may be easier to assess than older TGD populations. Nevertheless, the limited available data that compares younger to older TGD individuals suggests that those who are younger experience more psychological distress and are more likely to engage in non-suicidal self-injury [57, 58], indicating that younger age may provide a window for intervention. The pattern of findings might also differ if the sample were more racially diverse; relative to white TGD individuals, TGD individuals of color are more likely to be unemployed, live in poverty, have HIV, and face discrimination when accessing healthcare [7, 59]; from a minority stress perspective, all of these factors may contribute to increased psychological distress.

In addition to sample and setting related limitations, our analyses were restricted by the data that were available within the electronic medical record (EMR). First, we could only examine differences among the three existing gender options within the system, which may restrict clinically meaningful findings [60]. Second, though behavioral health and substance use appointments were distinct entities within the medical record, it was unclear which psychological concerns were addressed in behavioral health appointments and which SUDs were being treated in substance use appointments. Third, there may have been a lack of consistency in treatment referral practices across providers, and we could not associate the ePRO scores with a specific behavioral health referral or appointment. Fourth, although scores above certain thresholds on the ePRO measures are suggestive of likely depression, anxiety, and substance use disorders, the EMR does not contain thorough provider-level assessments that confirm these diagnoses. Relatedly, EMR data do not capture the psychological or substance use treatment that patients may have received outside of the community health center, as patients may not have had access to in-house treatment (i.e., long wait times and/or other barriers) or may have been engaged in treatment elsewhere. Additionally, because we captured all behavioral health and substance use visits that took place during the three-year period, patients may have already been in treatment before completing the ePRO measures. Lastly, length of time affiliated with the community health center was not accounted for in the analyses that examined engagement with behavioral health and substance use services across gender category, as this data was not available. As such, patients who initiated care during the three-year period may have had less time to engage with treatment and therefore less opportunity to attend sessions relative to patients who initiated primary care prior to 2015.

The high risk for depression, anxiety, and substance use disorders among TGD individuals and non-binary individuals in particular underscores the importance of assessment. Gender-related data are not systematically collected in clinical and research contexts [61], which likely contributes to the lack of attention paid to the specific mental health needs of TGD subgroups. Others, including the National Academies of Sciences, Engineering, and Medicine, have also expressed the need for standardized gender (and sexual orientation) data [62], as national surveys have failed to assess both sex assigned at birth and gender, unlike the current study. Indeed, if gender (including assigned sex at birth and current gender) is not thoroughly, competently, and affirmingly assessed across large healthcare systems, in electronic medical records, and in randomized clinical trials, important mental health differences that may be related to gender minority stress could be attributed to other factors or missed entirely. Routine collection of gender and sex identity data will not only advance our understanding of mental health risk and associated treatment disparities across TGD subpopulations, it will also help us determine which unique resilience factors can be targeted and leveraged in treatment [3].
